# Supplementary material for: Causal Relationship between Aging and Anorexia Nervosa: A White-Matter-Microstructure-Mediated Mendelian Randomization Analysis
Source: Biomedicines. 2024 Aug 16;12(8):1874. doi: 10.3390/biomedicines12081874 (PMC11351342; doi:10.3390/biomedicines12081874)
Supplement: Supplementary file 1 [file biomedicines-12-01874-s001.zip › Supplementary Figure/Figure S4/Singe SNP plot of MR analysis for TL to ubm-b-2103.pdf]

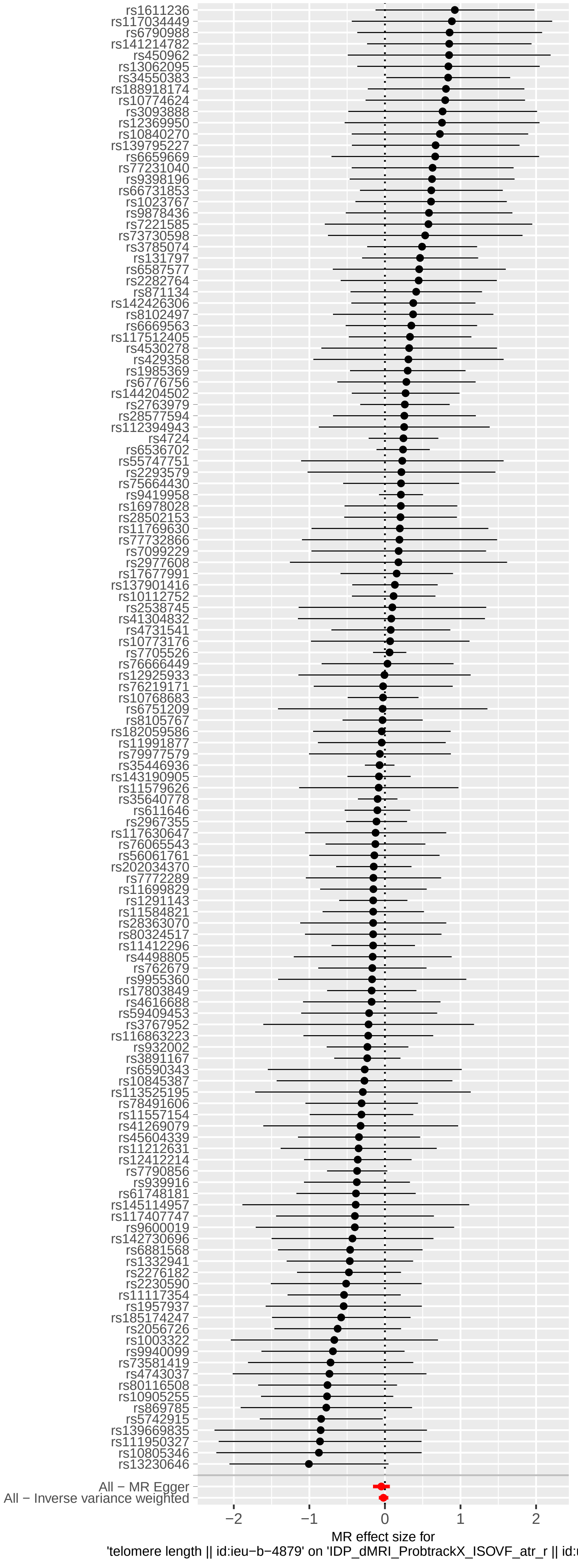
rs1611236
rs117034449
rs6790988
rs141214782
rs450962
rs13062095
rs34550383
rs188918174
rs10774624
rs3093888
rs12369950
rs10840270
rs139795227
rs6659669
rs77231040
rs9398196
rs66731853
rs1023767
rs9878436
rs7221585
rs73730598
rs3785074
rs131797
rs6587577
rs2282764
rs871134
rs142426306
rs8102497
rs6669563
rs117512405
rs4530278
rs429358
rs1985369
rs6776756
rs144204502
rs2763979
rs28577594
rs112394943
rs4724
rs6536702
rs55747751
rs2293579
rs75664430
rs9419958
rs16978028
rs28502153
rs11769630
rs77732866
rs7099229
rs2977608
rs17677991
rs137901416
rs10112752
rs2538745
rs41304832
rs4731541
rs10773176
rs7705526
rs76666449
rs12925933
rs76219171
rs10768683
rs6751209
rs8105767
rs182059586
rs11991877
rs79977579
rs35446936
rs143190905
rs11579626
rs35640778
rs611646
rs2967355
rs117630647
rs76065543
rs56061761
rs202034370
rs7772289
rs11699829
rs1291143
rs11584821
rs28363070
rs80324517
rs11412296
rs4498805
rs762679
rs9955360
rs17803849
rs4616688
rs59409453
rs3767952
rs116863223
rs932002
rs3891167
rs6590343
rs10845387
rs113525195
rs78491606
rs11557154
rs41269079
rs45604339
rs11212631
rs12412214
rs7790856
rs939916
rs61748181
rs145114957
rs117407747
rs9600019
rs142730696
rs6881568
rs1332941
rs2276182
rs2230590
rs11117354
rs1957937
rs185174247
rs2056726
rs1003322
rs9940099
rs73581419
rs4743037
rs80116508
rs10905255
rs869785
rs5742915
rs139669835
rs111950327
rs10805346
rs13230646
All − MR Egger
All − Inverse variance weighted
−2
−1
0
1
2
MR effect size for
'telomere length || id:ieu−b−4879' on 'IDP_dMRI_ProbtrackX_ISOVF_atr_r || id:
